# Bronchopulmonary dysplasia to predict neurodevelopmental impairment in infants born extremely preterm

**DOI:** 10.1038/s41390-024-03601-w

**Published:** 2024-10-24

**Authors:** Olivier Baud, Philippe Lehert, On behalf of the PREMILOC study group

**Affiliations:** 1 Department of Neonatal Medicine, Cochin-Port Royal Hospital, University Paris Cité, Paris, France; 2 University of Geneva, Geneva, Switzerland; 3 Faculty of Medicine, University of Melbourne, Melbourne, Australia; 4 Faculty of Economics School of Management, University of Louvain, Louvain, Belgium

## Abstract

**Background:**

Bronchopulmonary dysplasia (BPD) in extremely low gestational age neonates (ELGANs) was associated with neurodevelopmental impairment (NDI). However, the best endpoint of BPD assessment to predict subsequent NDI remains unclear.

**Methods:**

We re-analyzed the PREMILOC trial, previously designed to test the effect of prophylactic hydrocortisone on survival without BPD at 36 weeks of postmenstrual age (BPD_W36_) in ELGANs, to compare predictive models of NDI considering baseline characteristics, respiratory course up to and BPD status at 36 or 40 weeks of postmenstrual age (BPD_W36_/BPD_W40_).

**Results:**

Among 404/519 (77.8%) infants enrolled in the trial alive at 2 years of age, all neurocognitive scores were available for 302 (74.8%) patients. Gestational diabetes and sex were identified as the only statistically significant baseline predictors of NDI. Adding BPD_W40_ to this baseline model was found to be superior to predict NDI compared to BPD_W36_, leading to a mean difference of the developmental quotient of −6.7 points (95% confidence interval: −10.0 to −3.50, *P* < 0.001). The prophylactic hydrocortisone treatment effect on survival without BPD_W40_ was found to be highly significant (OR = 2.08 [95% confidence interval: 1.36 to 3.17], *P* < 0.001).

**Conclusions:**

These data suggest a better accuracy of BPD_W40_ to predict NDI in ELGANs, an important finding for future clinical trials and research in drug development.

**Registration numbers:**

EudraCT number 2007-002041-20, ClinicalTrial.gov number, NCT00623740.

**Impact:**

- The best endpoint to assess BPD as a surrogate to predict neurocognitive impairment in infants born extremely preterm remains unclear.
- This study strongly suggests a better discriminative value of BPD as assessed at 40 weeks of postmenstrual age (instead of 36 weeks) to predict neurocognitive impairments at 2 years of age in children born extremely preterm.
- This study supports the switch up to 40 weeks of the primary outcome chosen in future clinical trials designed to prevent BPD.
- Our data also provide evidence of the beneficial effect of HC on preventing BPD at full-term equivalent age.

## Introduction

Bronchopulmonary dysplasia (BPD) is the most common serious complication in infants delivered extremely low gestational age neonates (ELGANs)^1^ and its severity was found to worsen their neurodevelopmental impairment (NDI).^2^ Systemic inflammation occurring during the perinatal and neonatal period is considered a common risk factor for both the developing lung and the developing brain.^3,4^ While there are few therapeutic options to prevent BPD, prophylactic low-dose hydrocortisone (PHC) has been reported to improve survival without BPD in ELGANs.^5–7^ Several definitions of BPD have been proposed based on improved survival, changes in the management of ELGANs, and new modalities of respiratory support.^8,9^ While BPD definitions show similar accuracy in predicting long-term NDI,^10^ the best endpoint of postmenstrual age (PMA) to assess BPD as a surrogate for NDI in infants born extremely preterm remains to be investigated.

The endpoint to define BPD has been commonly set up at 36 weeks of postmenstrual age (BPD_W36_) for decades. However, there is no consensus that this timepoint is the most predictive of chronic lung disease or the most appropriate for evaluating the clinical impact of new drugs or therapeutic strategies.^11^ Indeed, the BPD_W36_ definition remains an imperfect surrogate for predicting chronic respiratory morbidity in childhood and adolescence.^12^ In contrast to the early BPD_W36_ endpoint, BPD determined at 40 weeks PMA (BPD_W40_) has been shown to better identify infants with severe respiratory morbidity at 18–21 months corrected gestational age.^13^ However, the predictive value of BPD_W40_ assessment regarding the long-term NDI has never been investigated in infants born very preterm.

The primary objective of this post-hoc analysis of the PREMILOC trial was to sequentially evaluate and compare prognostic models based on baseline characteristics, treatment options, BPD_W36_, and finally BPD_W40_ to predict neurocognitive impairment in ELGANs at 2 years of age. Our secondary objectives were to reassess HC treatment effect on survival without BPD_W40_ and to examine its impact on neurocognitive outcomes.

## Patients and Methods

### Study population and database

We conducted a post-hoc analysis of the PREMILOC trial, which was a double-masked, placebo-controlled, multicenter randomized trial that enrolled extremely preterm infants delivered from 21 perinatal centers in France before 28 weeks of gestation. Eligible infants were randomly assigned to receive either prophylactic low-dose hydrocortisone or placebo within 24 h after birth and for 10 days. The trial was approved by the national ethics committee (Comité de Protection des Personnes (CPP), Ile-de-France II, Necker), the French National Drug Safety Agency (ANSM, EudraCT number 2007-002041-20), and the French data protection authority (Commission Nationale de l’Informatique et des Libertés; CNIL). Prior to randomization, written informed consent was obtained from the parents of all eligible infants before. The trial was registered at ClinicalTrials.gov (NCT00623740) before the first patient was enrolled. The study protocol and main analyses have been previously reported.^6,14,15^

### Primary outcome

The main endpoint of the present study was the Revised Brunet-Lezine (RBL) test, a psychomotor developmental scale that evaluates 4 domains of development, including gross motor function, fine motor function and visuospatial coordination, language, and sociability in children aged 2 to 30 months (a short description of RBL scale is provided in Supplementary materials).^16^ A global developmental quotient score results from the combination of the RBL sub-scores, with a mean norm of 100 (SD, 15).

### Concomitant treatments calculation

Concomitant treatments, considered to be related to respiratory status of the infants in line with a consensus from the investigators of PREMILOC trial are as follows: selective steroids treatment after the end of prophylactic HC treatment period (day 10) (supplemental HC, betamethasone, solumedrol), diuretics (bumetanide, hydrochlorothiazide, furosemide, spironolactone). Concomitant treatments exposure was expressed as the total duration in days of any of these drugs.

### Statistical analyses

#### Primary analysis

**Fig. 1:**
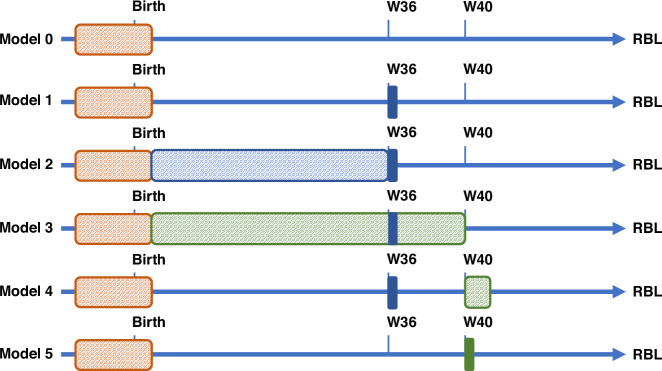
Graphical representation of models used to predict 2-year revised Brunet-Lezine (RBL) scores.

A statistical analysis plan was predefined by the authors and locked before processing the analysis. Our primary objective was to develop the best predictive model for NDI at 2 years of age. Potential predictors were all the available baseline characteristics in particular initial severity of the neonate’s conditions, socio-demographic status, maternal ethnicity, and type and duration of the respiratory support (ventilation and concomitant treatments) and adverse events that occurred before hospital discharge including sepsis, patent ductus arteriosus (medical treatment or surgical ligation), perinatal asphyxia, necrotizing enterocolitis. We compared different models characterized by binary or continuous measurements at different time points (Fig. 1) :

- Model 0: Predictive model limited to baseline characteristics only.

- Model 1: BPD_W36_ gold standard predictor was added to the significant predictors of Model 0, and considered as the reference for the subsequent models.

- Model 2: Instead limiting the measurement at 36 weeks of PMA, we tested the additive predictive value of the continuous duration of the mechanical ventilation, continuous positive airway pressure (CPAP), supplemental O_2_ duration, and concomitant medication from birth until 36 weeks.

- Model 3: Since respiratory status at 40 weeks of PMA was considered more relevant than that at 36 weeks PMA,^12,13^ the additive predictive value of continuous measurements of the same covariates measured from baseline until 40 rather than 36 weeks of PMA was examined.

- Model 4: As done in model 3, the additive predicted value of the same continuous measurements was restricted during the 40^th^ week of PMA, rather than from birth.

- Model 5: Binary classification of BPD_W40_ was defined as the need for respiratory support (mechanical, CPAP, or supplemental oxygen) or concomitant medication (including diuretics and steroids) associated with chronic lung disease, during the 40^th^ week of PMA.

For each model, we used a linear model using a stepwise selection strategy optimizing AIC (Akaike Information Criteria).^17^ Due to the known limitations of this strategy,^18^ sensitivity analyses were replicated using a backward strategy for the RBL general score and 4 sub-scores. Each model was compared with the reference model 1, in assessing the significance of the change in the R2 coefficient (ΔR2 test) and comparing the Bayes Information Criterion (BIC).

### Secondary analyses

We reassess the HC effect, already demonstrated on BPD_w36_,^15^ on the best model developed here above. The multiple interdependent relations in which variables are simultaneously dependent and independent necessitated a Structural Equations Modelling SEM approach,^19^ implemented in the Lavaan R Package^20^ and Ωnyx.^21^ A pre-specified structural scheme was tested (χ2 test comparing the observed and induced covariances), and the effect of HC on NDI was tested as a direct and indirect effect through a mediation process.^22^

All statistical tests were performed at a two-sided significance level of 0.05. The statistical analyses were carried out using R release 4.1.0 for Windows (R Core team, 2021).

## Results

### Description of the population

Of the 523 extremely preterm infants recruited in the PREMILOC trial, a total of 519 infants were included for the current post-hoc analyses, as the parents of one infant in the HC group and three infants in the placebo group withdrew their consent. Among these 519 infants, 404 (77.8%) were alive (including 197 in the placebo group and 207 in the HC group) and out of these, a total of 377 were evaluated at a median corrected age of 22 months. All RBL scores and subscores were available for 302 patients. The 75 remaining patients, who were assessed only by standardized neurological examination were excluded from the present analysis. The baseline data and neonatal characteristics of these children and their RBL scores at 2 years of age are summarized in Table 1.

**Table 1:** Prenatal, baseline, neonatal characteristics and 2-year outcomes of surviving infants included in the analysis.

Variable	Studied population (*n* = 302)
**Prenatal variables**	
Mother Age (years)	
Median (IQR)	30.3 (26.8–35.4)
Multiple pregnancy	98 (32.5%)
Gestational diabetes	16 (5.3%)
Gestational hypertension	31 (10.3%)
Chorioamnionitis	151 (50.0%)
Premature rupture of the membranes > 24 h	93 (30.8%)
Antenatal steroids	289 (95.7%)
Epidural analgesia	142 (47.0%)
General anesthesia	36 (11.9%)
Tocolysis	204 (67.5%)
Antenatal antibiotics	215 (71.2%)
Cesarean section	140 (46.4%)
**Neonatal variables**	
Birthweight (g)	
Median (IQR)	873 (755–985)
Gestational age (days)	
Median (IQR)	26.6 (25.8–27.4)
Female sex	144 (47.7%)
Intra Uterine Growth Restriction	26 (8.6%)
Respiratory support baseline	
- Mild	115 (38.1%)
- Moderate	157 2.0%)
- Severe	30 (9.9%)
**Clinical respiratory variables up to discharge**	
BPD at 36 weeks PMA	90 (29.8%)
BPD at 40 weeks PMA	79 (26.2%)
Mechanical ventilation (day)	
Median (IQR)	22 (2–37)
Supplemental O_2_ (day)	
Median (IQR)	62 (47–81)
Concomitant treatments (day)	
Median (IQR)	53 (26–68)
Number of patients exposed to steroids at 40 weeks PMA	14 (4.6%)
Number of patients exposed to diuretics at 40 weeks PMA	14 (4.6%)
Hospital stay (day)	
Median (IQR)	89 (76–111)
***Neurocognitive assessment at 2 years*** *Median RBL scores (IQR)*	
Global score	92 (85–100)
Motor subscore	102 (91–111)
Coordination	92 (80–101)
Language	85 (75–95)
Social	96 (85–111)

IQR means interquartile range.

Respiratory support baseline:

-mild: non-invasive ventilation with FiO_2_ < 30%.

-moderate: respiratory mechanical ventilation with FiO_2_ < 30%.

-severe: mechanical ventilation with FiO_2_ ≥ 30%.

*IQR* interquartile range.

*RBL* Revised Brunet-Lezine scores.

### Comparisons of the models studied (Table 2)

Model 0 restricted to baseline variables: stepwise, confirmed by backward elimination, restricted the significant predictors to gestational diabetes (−8.2, 95% confidence interval [CI]: −14.7 to −2.1, *P* < 0.014) and sex (6.2, 95% CI: 3.3–9.1, *P* < 0.001) with a coefficient of determination of R^2^ = 0.079 (*P* < 0.001). These associations were confirmed for each subscore of the RBL (Supplementary Table 1).

**Table 2:** Comparison of linear models to predict revised Brunet-Lézine global score in 2-year infants born extremely preterm according to the endpoints considered.

	Model 0		Model 1		Model 2		Model 3		Model 4		Model 5	
**Variable**	Baseline model		+ BPD_W36_ only		+ clinical course up to 36 weeks of PMA including BPD_W36_		+ clinical course up to 40 weeks of PMA		+ clinical status at 40 weeks of PMA		+ BPD_W40_ only	
	MD (95% CI)	*p*-value	MD (95% CI)	*p*-value	MD (95% CI)	*p*-value	MD (95% CI)	*p*-value	MD (95% CI)	*p*-value	MD 95%CI	*p*-value
**Female sex**	6.2 (3.3, 9.1)	< 0.001	5.3 (2.4, 8.3)	< 0.001	5.3 (2.4, 8.3)	< 0.001	5.8 (2.9, 8.7)	< 0.001	8.9 (5.1, 12.8)	< 0.001	5.35 (2.5, 8.2)	< 0.001
**Gestational diabetes**	−8.2 (−15, −2.1)	0.014	−7.6 (−14.1, −1.1)	0.020	−7.7 (−14.2, −1.2)	0.020	−7.8 (−14.3, −1.4)	0.018	−11.1 (−19.7, −2.5)	0.012	−7.6 (−14.0, −1.2)	0.020
**BPD**_**W36**_			−4.7 (−7.9, −1.4)	0.004	−4.78 (−8.0, −1.5)	0.004						
**Mechanical ventilation (day)**							−0.09 (−0.15, −0.02)	0.003				
**Concomitant treatments (day)**							−0.03 (−0.08, −0.00)	0.045				
**Supplemental O**_**2**_ **(day)**									−0.91 (−1.43, −0.40)	< 0.001		
**Hospital stay (day)**												
**BPD**_**W40**_											−6.7 (−10, −3.5)	< 0.001
R2	0.079		0.101		0.101		0.121		0.102		0.120	
**DR2**	**-**		***p*** = **0.004 (vs model 0)**		***p*** = **0.994 (vs model 1)**		***p*** = **0.042 (vs model 1)**		***p*** = **0.046 (vs model 1)**		***p*** = **0.045 (vs model 1)**	

*MD* Mean difference of revised Brunet-Lézine global score, *CI* confidence interval, *PMA* Postmenstrual age.

BPD_W36_ / BPD_W40_: bronchopulmonary dysplasia assessed at either 36 or 40 weeks of postmenstrual age.

Blank cells correspond to variables included but not retained in the stepwise regression.

Green cells correspond to variables non included in the stepwise regression.

R2 Coefficient of determination ΔR2: Significance of the change of the determination coefficient compared with another model.

Model 1 added BPD_W36_ to the previous model: the effects of female sex and gestational diabetes are confirmed with a significant effect of BPD_W36_ (−4.7, 95% CI: −7.9 to −1.4, *P* < 0.004) and an improved coefficient of determination (R^2^ = 0.104) compared to Model 0 (*P* < 0.004).

Model 2 added duration of ventilatory support, hospital stay, and concomitant medication up to 36 weeks of PMA to Model 1. None of these additional variables provided a significant improvement compared to Model 1.

Model 3 based on Model 1, tested the additive predictive value of the same variables but measured up to 40 weeks instead of 36 weeks of PMA. The baseline covariates of Model 1 were confirmed with the significant effect of duration of mechanical ventilation and concomitant medication. Coefficient determination of Model 3 was found to be superior to Model 1 (R^2^ = 0.121, ΔR^2^ *P* < 0.042).

Model 4 complemented Model 1 with the same variables restricted during week 40. The effect of O_2_ supply was confirmed for all subscores but with a lower coefficient of determination R^2^ = 0.102 than in Model 3.

Model 5 examined the additive predicted value of the BPD_W40_, instead of BPD_W36_, as defined as a subject with either mechanical ventilation, supplemental O_2_ oxygen supply or concomitant treatment recorded at week 40. We found a highly significant effect of the BPD_W40_ binary end point both for global RBL score (−6.7, 95% CI: −10.0 to −3.5, *P* < 0.001) and for all RBL subscores (Supplementary Table 2), with a coefficient of determination R^2^ = 0.120 similar to Model 3 and superior to model 1. This model was therefore considered as the best. Interestingly, lat onset sepsis, patent ductus arteriosus (requiring medical treatment or surgical ligation), perinatal asphyxia, and necrotizing enterocolitis as postnatal complications were not significantly associated with neuromotor development when added to this model (Supplementary Table 3).

### Effect of prophylactic HC on survival without BPD at 40 weeks of PMA

We tested the beneficial effect of HC on survival without BPD by using BPD_W40_ endpoint (instead of BPD_W36_ as already published^14^). We found success proportion of 136/264 (51.5%) vs 168/255 (65.9%) in the HC group compared to the placebo group. Adjusting for our predictive model^7^ (Table 3) resulted into a HC treatment effect estimated by an odds ratio of 2.08 (95% CI [1.36–3.17], *p* < 0.001), equivalent to a Ratio Risk of RR = 1.35 (95% CI, 1.15–1.52), an absolute risk difference of 17.4% (95% CI, 7.6%–26.1%), and a number needed to treat of 5.74 (95% CI, 3.84–13.11).

**Table 3:** Logistic mixed model regression to assess the effect size of early low-dose hydrocortisone (HC) on survival without BPD at 40 weeks of PMA, adjusted on the patient’s probability of BPD-free survival depending on baseline predictors only.

	Odds ratio	95% CI	*p*-value
Overall Estimate	0.633	0.514–0.737	0.028
Multiple pregnancy	0.611	0.394–0.949	0.029
Gestational age at birth	1.511	1.155–1.978	0.003
Birthweight	1.005	1.003–1.007	< 0.001
Female sex	1.931	1.251–2.977	0.003
RSB-moderate	0.563	0.339–0.935	0.027
RSB-severe	0.181	0.094–0.347	< 0.001
HC treatment	2.077	1.362–3.171	< 0.001

RBS means respiratory support baseline:

-moderate: respiratory mechanical ventilation with FiO_2_ < 30%.

-severe: mechanical ventilation with FiO_2_ ≥ 30%.

### Effect of prophylactic HC on NDI

**Fig. 2:**
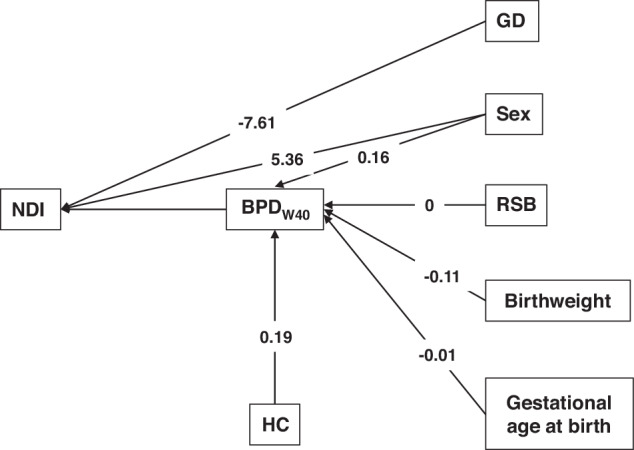
Structural Model summarizing the effects of baseline conditions, BPD at 40 weeks of PMA (BPD_W40_) and early hydrocortisone treatment (HC), validated by the goodness of fit test (χ2 = 1.411, df = 4, *p* = 0.842). The effect of each direct path is reported. The influence of an independent variable on a dependent variable is depicted by a line with a directional arrow, and the effect or regression coefficient of each independent variable is reported at the side of each arrow. The HC effect was investigated through a mediation process: its effect on BPD_W40_ (0.19 ± 0.045, *P* < 0.001) combined with the BPD_W40_ effect on RBL (6.76 ± 1.69, *P* < 0.001) results into an indirect significant effect (1.40 ± 0.46 [0.48, 2.32], *P* = 0.002) of HC mediated by BPD_W40_. No additional effect of HC is concluded due to the non-significant direct effect (*P* = 0.205) of HC on RBL. RSB respiratory support at baseline. GD Gestational diabetes

Although HC has not been associated with a statistically significant difference in neurological development,^14^ there is evidence that the neurological development of children born at 24 and 25 weeks gestation exposed to HC is better.^16^ As shown here above, HC has a significant effect on BPD_W40_ which in turn affects NDI. This sequential effect HC → BPD_W40_ → NDI was examined using a structural model (Fig. 2) summarizing our previous findings: baseline variables and HC treatment affect BPD (Table 3 and ref. ^23^), while predictors of RBL are sex of the newborn, gestational diabetes, and BPD_W40_ (Table 2). Model fit was accepted (χ2 = 1.411, df=4, *P* = 0.842) and all paths were found to be significant. The effect of HC on BPD_W40_ (0.19 ± 0.045, *P* < 0.001) combined with the effect of BPD_W40_ on RBL (7.23 ± 1.69, *P* < 0.001) resulted in an indirect significant effect (1.4 ± 0.46, 95%CI: 0.48–2.32, *P* = 0.002) of HC on RBL.

## Discussion

There is no consensus that BPD_W36_ is the best predictor of chronic pulmonary insufficiency^11^ or NDI. Our data strongly suggest that BPD_W40_ has better accuracy in predicting NDI in ELGANs. In addition, we further confirmed the benefits of HC treatment on survival without BPD_W40_, even better than previously reported for BPD_W36_.^24^

Of the baseline covariates, only the sex of the newborn and diabetes during pregnancy were found to be significant predictors of NDI. These findings strongly differ from our previous prognosis model to predict survival without BPD in which birthweight, gestational age, multiple pregnancy, or ventilatory support within hours after birth, but not sex were found to be significant.^19^ This can be easily explained by the fact that most of these variables were closely related to neonatal death, which was excluded in the present study. Sex was a well-known variable associated susceptibility to brain injury in preclinical studies,^20^ which has traditionally been attributed to the protective effects of estrogens.^21^ Similarly, male sex in human neonates has been associated with an increased risk of NDI in infants born very prematurely, compared to females. This increased risk may be modulated by obstetric risk factors,^22^ particularly severe fetal growth restriction,^23^ and neonatal complications.^25^ *In utero* exposure to insulin-dependent diabetes mellitus was associated, in ELGANs, with a higher risk of small head circumference but not with NDI.^26^ Nevertheless, infants exposed to gestational diabetes were also found to have a lower intelligence quotient and an increased risk of autism spectrum disorders.^27^

BPD_W36_ significantly improved the accuracy of predicting NDI, confirming data also reported at 10 years of age,^28^ with no additional value provided by continuous values of concomitant medication, ventilatory support, and duration of hospitalization until 36 weeks of PMA. Conversely, using 40 weeks of PMA instead of 36 weeks as the final endpoint for these variables provided a more accurate prediction of NDI. A later endpoint is usually characterized by better regulated respiratory control and advanced feeding maturation with gastric tube removal in ELGANs. All these improvements allow better identification of infants with severe parenchymal disease that persists up to term equivalent of age, itself related to tissue/systemic inflammation detrimental to the developing brain.^29^ While the dichotomous outcome BPD_W40_ *vs* no BPD was reported here as the best outcome for predicting NDI, we also found that a composite outcome including medication for respiratory support, supplemental oxygen, and ventilatory support during week 40 of PMA yielded a similar determination, as previously suggested by Steinhorn et al.^12^ Similar findings were also reported by the Canadia Neonatal Network to predict respiratory morbidity at 18-21 months of corrected age.^13^ When considering concomitant medications separately, we also found that both diuretics and steroids were predictive of neuro-motor development, with steroids performing slightly better. However, the best coefficient of determination was obtained by summing the overall concomitant medication exposure.

The size effect of HC to prevent BPD is enhanced when 40 weeks of PMA is used as the main endpoint with an NNT of 5.7, compared with 12 as reported in the parent analysis calculated at 36 weeks of PMA.^24^ HC could therefore prevent later stage BPD defined at full equivalent of age. Furthermore, Structural Equation Modelling provided evidence for the benefit of HC on NDI: HC reduces BPD_40_, which in turn is associated with a decrease in NDI, and this indirect HC → BPD_W40_ → NDI relationship results in a significant effect of HC on NDI.

This study has several limitations. First, the lack of follow-up using RBL in 25% of infants may affect the accuracy of each model. Second, the estimated coefficients for estimating the effect of respiratory supply or concomitant medication can vary among centers, depending on the local standards and protocols. Third, the Bayley Scales Of Infant and Toddler Development are commonly used in the assessment of neurological development in very preterm infants, the RBL test was used in the present study. Indeed, we chose this test because it was adjusted on the basis of a large sample of 1055 French children born at term and is routinely used in studies conducted in French-speaking countries. Finally, we were not able to assess the predictive value of BPD_W40_ in infants aged 5 years, because 5-y outcomes were only assessed in a minority of enrolled subjects.^30^

This study highlights the association between BPD and later neurocognitive outcomes in ELGANs by demonstrating that later endpoints assessing BPD may be more predictive of NDI. These findings have implications for both clinical practice and research, as they encourage a switch to 40 weeks as the preferred BPD endpoint in future clinical trials. Our data also demonstrate a beneficial effect of HC to prevent BPD at 40 weeks of PMA, which in turn is associated with a decrease in NDI. This suggests that long-term neurological development should be considered not only as a safety outcome but also as a pre-specified secondary endpoint to assess the superiority of early HC compared to placebo.

## Supplementary information

Appendix

Supplementary materials

## Data Availability

The data supporting the findings of this study are available from Assistance Publique Hôpitaux de Paris; however, access to these data is subject to certain restrictions as they were used under license for the current study. Therefore, the data is not publicly available. Nonetheless, researchers may request access to the data from the authors, subject to approval from Assistance Publique Hôpitaux de Paris.
