# Supplementary material for: Bronchopulmonary dysplasia to predict neurodevelopmental impairment in infants born extremely preterm
Source: Pediatr Res. 2024 Oct 24;97(7):2436–42. doi: 10.1038/s41390-024-03601-w (PMC12279537; doi:10.1038/s41390-024-03601-w)
Supplement: Supplementary file 1 — Appendix [file 41390_2024_3601_MOESM1_ESM.pdf]

Appendix

PREMILOC trial Study Group members (all in France)

Valérie Biran, PhD, Aline Rideau Batista-Novais, PhD, Caroline Farnoux, MD, Sophie Soudée, MD, Laure Maury, MD: University hospital Robert Debré, APHP, Paris

Michèle Granier, MD, Florence Lebail, MD : General Hospital, Corbeil-Essonnes

Duksha Ramful, MD, Sylvain Samperiz, MD ; Regional Hospital Saint-Denis, La Réunion

Alain Beuchée, PhD, Karine Guimard, MD : University Hospital, Rennes

Fatima El Moussawi, MD, Pascal Boileau, PhD, Florence Castela, MD : General Hospital, Poissy

Claire Nicaise, MD, Renaud Vialet, MD : University Hospital, Hôpital Nord, APHM, Marseille

Pierre Andrini, MD, Thierry Debillon, PhD : University Hospital, Grenoble

Véronique Zupan-Simunek, MD, Hasinirina Razafimahefa, MD : University Hospital Antoine Béclère, APHP, Paris

Anne Coursol, MD, Saïd Merbouche, MD : General Hospital, Pontoise

Pascal Bolot, MD, Jean-Marc Kana, MD : General Hospital Saint-Denis

Julie Guichoux, MD, Olivier Brissaud, PhD: University Hospital, Bordeaux

Gérard Thiriez, PhD, Olivier Schulze, MD, University Hospital Besançon

Mickael Pomedio, MD, Patrice Morville, PhD: University Hospital, Reims

Thierry Blanc, MD, Stéphane Marret, PhD: University Hospital, Rouen

Bernard Guillois, PhD, Cénéric Alexandre, MD, University Hospital, Caen

Stéphane Le Bouëdec, MD, Bertrand Leboucher, MD: University Hospital, Angers

Umberto Simeoni, PhD, Valérie Lacroze, MD: University Hospital La Conception, APHM, Marseille

Pierre Kuhn, PhD, Stéphanie Litzler-Renaud, MD: University Hospital Strasbourg

Elodie Zana-Taïeb, MD, Pierre-Henri Jarreau, PhD: University Hospital Cochin-Broca-Hôtel Dieu, APHP, Paris

Sylvain Renolleau, PhD, Virginie Meau-Petit, MD : University Hospital Armand Trousseau, APHP, Paris

Gilles Cambonie, PhD: University Hospital Montpellier.

Corinne Alberti, PhD Aurélie Bourmaud, PhD : Unit of Clinical Epidemiology, Assistance Publique-Hôpitaux de Paris, CHU Robert Debré, University Paris Diderot, Sorbonne Paris-Cité, Inserm U1123 and CIC-EC 1426, Paris.
