# Supplementary material for: Bronchopulmonary dysplasia to predict neurodevelopmental impairment in infants born extremely preterm
Source: Pediatr Res. 2024 Oct 24;97(7):2436–42. doi: 10.1038/s41390-024-03601-w (PMC12279537; doi:10.1038/s41390-024-03601-w)
Supplement: Supplementary file 2 — Supplementary materials [file 41390_2024_3601_MOESM2_ESM.pdf]

Supplementary Materials

Short description of Revised Brunet Lezine scale

The revised Brunet-Lézine allows to perform reliable, simple and rapid child development of children from 2 to 30 months and identify any discrepancies between them and an age group representative of the French population.

Numerous technical changes have been made to this revised version. The material has been changed and updated to bring it up to date and to make it more attractive to children. Elements of the old version have been reviewed, changed or deleted and new elements have been developed.

However, much of the original content and structure of the Brunet-Lézine has been retained. The basic functions have been retained and improved, namely :

- a standardized observation situation of the child
- a quick and easy application
- use of materials that arouse the child's interest
- minimal influence of the examiner thanks to a clear presentation and strict scoring principles
- calculation of age and development quotients

The tests are divided into 4 sections:

- motor skills or posture
- Oculo-manual coordination control
- Language skills
- Social skills

10 tasks are proposed for each age level, but the proportion of each varies from one area to another, reflecting the child's learning progress.

The analysis of the results makes it possible to calculate partial development ages and ratios in the 4 areas studied, as well as an overall development age and ratio.

<u>References</u>:

Brunet O, Lézine I. Le développement psychologique de la première enfance, présentation d'une échelle française pour examen des tout petits. Population. 1952;7(1):162.Google ScholarCrossref

Lézine I. Le développement psychomoteur des jeunes prématurés. Etud Neo-natales (Paris). 1958;7(1):1-50.Google Scholar

Josse D. Brunet-Lézine Révisé: Echelle de Développement Psychomoteur de la Première Enfance. Paris, France: Etablissements d'applications Psychotechniques; 1997.

Supplementary Tables

Variable	RBL global MD (95% CI)	RBL global p-value	RBL motor MD (95% CI)	RBL motor p-value	RBL coordination MD (95% CI)	RBL coordination p-value	RBL language MD (95% CI)	RBL language p-value	RBL social MD (95% CI)	RBL social p-value
**Gestational diabetes**	-8.2 (-14.8 to -1.7)	0.014	-5.6 (-15.3 to 4.1)	0.258	-6.0 (-13.5 to 1.5)	0.117	-8.7 (-16.8 to -0.6)	0.036	-13.0 (-21.7 to -4.3)	0.004
**Female sex**	6.2 (3.3 to 9.1)	<0.001	4.2 (-0.2 to 8.5)	0.061	4.8 (1.4 to 8.1)	0.006	7.9 (4.3 to 11.5)	<0.001	9.3 (5.4 to 13.2)	<0.001

Supplementary Table 1: Linear model (model 0) to predict revised Brunet-Lézine (RBL) scores at 2 years of age depending on baseline variables only.

MD: mean difference
CI: confidence interval

Variable	RBL global MD (95% CI)	RBL global p-value	RBL motor MD (95% CI)	RBL motor p-value	RBL coordination MD (95% CI)	RBL coordination p-value	RBL language MD (95% CI)	RBL language p-value	RBL social MD (95% CI)	RBL social p-value
**Gestational diabetes**	-7.6 (-14.0 to -1.2)	0.020	-	-	-	-	-8.0 (-15.9 to -0.1)	0.049	-12.3 (-20.8 to -3.7)	0.005
**Female sex**	5.4 (2.5 to 8.2)	<0.001	-	-	4.1 (0.7 to 7.5)	0.017	7.0 (3.4 to 10.6)	<0.001	8.2 (4.4 to 12.1)	<0.001
**BPD_{W40}**	-6.7 (-10.0 to -3.5)	<0.001	-6.5 (-11.5 to -1.6)	0.010	-5.1 (-1.4 to -8.7)	0.006	-7.6 (-11.7 to -3.6)	<0.001	-8.5 (-12.9 to -4.2)	<0.001

Supplementary Table 2: Linear model (model 5) to predict revised Brunet-Lézine (RBL) scores at 2 years of age depending on baseline variables and BPD assessed at 40 weeks of PMA.

MD: mean difference
CI: confidence interval
BPD: bronchopulmonary dysplasia
-: corresponds to variables included but not retained in the stepwise regression

	Effect	Se	95%CI	Pval
Overall Estimate	87.017	2.002	83.092,90.941	0.001
**Diabetes**	-8.161	3.239	-14.510,-1.812	0.012
**Female sex**	5.156	1.528	2.162,8.151	0.001
**BPD_{W40}**	6.451	1.783	2.956,9.946	0.001
**Late onset sepsis**	-0.188	1.734	-3.586,3.209	0.913
**Asphyxia**	-4.199	4.298	-19.622,7.224	0.295
**Necrotizing enterocolitis**	-0.622	2.950	-6.404,5.158	0.833
**Treated patent ductus arteriosus**	-1.741	1.923	-5.510,2.028	0.366
**Other adverse event**	-3.957	2.365	-8.592,0.678	0.095

Supplementary Table 3: Linear model (model 5) to predict revised Brunet-Lézine (RBL) scores at 2 years of age depending on baseline variables, neonatal complications and BPD assessed at 40 weeks of PMA.

BPD: bronchopulmonary dysplasia
